# Cycle-informed readiness: changes in training and nutrition throughout the female warfighter menstrual cycle

**DOI:** 10.1080/15502783.2025.2550194

**Published:** 2025-08-28

**Authors:** Tina Sergi, Jeffery Heileson

**Affiliations:** Walter Reed National Military Medical Center, Nutrition Services Department, Bethesda, MD, USA

**Keywords:** Military, performance, menstrual cycle, adaptation

## Abstract

**Background:**

The aims of this study were to 1) describe the influence of the menstrual cycle (MC) and hormonal contraceptive cycle (HC) symptoms on physical training; and 2) describe nutrition strategies to manage MC- and HC-related symptoms among active-duty female military personnel.

**Methods:**

A total of 162 active-duty females (age, 32.4 ± 6.9 years; branch, 91.4% Army; rank, 68.5% officer; HC user, 36.4%) completed a 50-question online survey. The survey included questions regarding demographics, HC use, MC- and HC-related symptoms, nutrition strategies to manage symptoms, and individual influence of the MC and HC on physical training and performance. Descriptive data for continuous and categorical variables were reported as mean ± SD and totals and percentages, respectively.

**Results:**

A majority of participants (*n* = 105, 64.8%) reported changing their training schedule due to MC/HC-related side effects in the past 12 months, whereas 31.5% (*n* = 51) did not change their training, 3.1% (*n* = 5) were unsure, and 0.6% (*n* = 1) did not respond. Among those who altered their training, 30.2% (*n* = 49) and 34.6% (*n* = 56) reported changing their training schedule 1–2 times and > 3 times, respectively. A range of side effects that influenced training were reported, with the most prevalent being pain in the stomach (*n* = 78, 48.2%), pain in the lower back (*n* = 77, 47.5%), and bloating (*n* = 70, 43.2%). Although a majority of the present sample reported changing their training due to their MC, only 18.5% (*n* = 30) reported planning their training according to their MC/HC cycle. In contrast, 79.6% (*n* = 129) did not plan their training around their cycle, and 1.9% (*n* = 3) were unsure. For nutrition strategies to combat MC/HC-related symptoms, the most common supplements included multivitamin (*n* = 50, 30.9%), magnesium (*n* = 37, 22.8%), and vitamin D (*n* = 36, 22.2%).

**Conclusions:**

The training regimens of female military personnel are influenced by the symptoms associated with the MC and HC. Future research is needed to determine if there are specific phases of the MC/HC (follicular, ovulatory, luteal) where training and performance are perceived to be most affected by the female warfighter, and where nutrition education can be provided to better manage symptoms and improve perceived performance.

